# Refractory Status Epilepticus in Genetic Epilepsy—Is Vagus Nerve Stimulation an Option?

**DOI:** 10.3389/fneur.2020.00443

**Published:** 2020-06-12

**Authors:** Nicola Specchio, Alessandro Ferretti, Nicola Pietrafusa, Marina Trivisano, Costanza Calabrese, Giusy Carfì Pavia, Alessandro De Benedictis, Carlo Efisio Marras, Luca de Palma, Federico Vigevano

**Affiliations:** ^1^Rare and Complex Epilepsy Unit, Department of Neuroscience, Bambino Gesù Children's Hospital, IRCCS, Member of European Reference Network EpiCARE, Rome, Italy; ^2^Neurosurgery Unit, Department of Neuroscience, Bambino Gesù Children's Hospital, IRCCS, Rome, Italy; ^3^Department of Neuroscience, Bambino Gesù Children's Hospital, IRCCS, Member of European Reference Network EpiCARE, Rome, Italy

**Keywords:** epilepsy, status epilepticus, vagal nerve stimulation (VNS), genetic epilepsies, treatment

## Abstract

Refractory and super-refractory status epilepticus (RSE, SRSE) are severe conditions that can have long-term neurological consequences with high morbidity and mortality rates. The usefulness of vagus nerve-stimulation (VNS) implantation during RSE has been documented by anecdotal cases and in systematic reviews; however, the use of VNS in RSE has not been widely adopted. We successfully implanted VNS in two patients with genetic epilepsy admitted to hospital for SRSE; detailed descriptions of the clinical findings and VNS parameters are provided. Our patients were implanted 25 and 58 days after status epilepticus (SE) onset, and a stable remission of SE was observed from the seventh and tenth day after VNS implantation, respectively, without change in anti-seizure medication. We used a fast ramp-up of stimulation without evident side effects. Our results support the consideration of VNS implantation as a safe and effective adjunctive treatment for SRSE.

## Introduction

Refractory and super-refractory status epilepticus (RSE, SRSE) are severe conditions that can have long-term consequences, including alteration of neuronal networks, neuronal injury, and high morbidity and mortality rates (1, 2). Conventional anti-seizure medications (ASMs) are ineffective, so off-label treatments are often administered (1).

Although vagus nerve stimulation (VNS) has been documented to reduce the occurrence and recurrence of status epilepticus (SE), debate continues on its value during RSE/SRSE. Single case reports and small case series of VNS implantation in RSE were included in a systematic review, where VNS implantation was associated with cessation in 76% of generalized and 26% of focal RSE (3). Also, VNS implantation has been reported to be associated with cessation of RSE/SRSE in 74% (28/38) of cases collected through a more recent systematic review of the literature (4). Also recently, effective VNS implantation during SRSE was reported in one patient with Lafora disease (5).

This report describes the electro-clinical findings and long-term outcome in two patients with genetic epilepsy who were successfully implanted with VNS during SRSE (an overview of clinical findings and VNS parameters is provided in Table 1). The publication of patient data from this investigational approach was approved by the local Ethics Committee.

**Table 1:** Overview of clinical findings and VNS parameters during SE.

	**Case #1**	**Case #2**
Age at RSE onset/Age of VNS implant	16 years	6 months
SE characterization	Super refractory myoclonic status (left arm and face and, rarely, right arm)	Repetitive focal to bilateral tonic-clonic seizures associated with apnea and cyanosis
ASDs tried during SE and before VNS implant	MDZ, LEV, LCM, VPA, TPM, PB, KET, methylprednisolone, IgIV	MDZ, PB, LEV, PER, KD (from day 9 to day 43 of SE)
Time between RSE onset and VNS implant	25 days	58 days
Day of VNS activation	Same day as VNS implantation	Same day as VNS implantation
Parameters of VNS activation	Intensity 0.25 mA, frequency 30 Hz, pulse width 500 microsec, duty cycle on-time 30 s, off-time 5 min	Intensity 0.25 mA, frequency 30 Hz, pulse width 500 microsec, duty cycle on-time 30 s, off-time 5 min
VNS amplitude titration	Up to 1.75 mA in 7 days (0.25 mA per day)	Up to 1.00 mA in 10 days (no more than 0.25 mA per day)
Improvement after VNS	SE stopped after setting the intensity at 1.75 mA at Day 7 (persistence of daily myoclonic seizures)	SE stopped after setting the intensity at 1.00 mA at Day 10 (persistence of weekly focal seizures)
VNS parameters at follow up	Increase of VNS intensity to 2.5 mA (frequency 30 Hz, pulse width 500 microsec, duty cycle on-time 30 s, off-time 5 min) led to a 50% reduction in myoclonic seizures	Four months after VNS implantation, seizures became more frequent and an attempt to further increase VNS intensity to 1.75 mA (frequency 30 Hz, pulse width 500 microsec, duty cycle on-time 30 sec, off-time 3 min) was ineffective
AE to VNS implant	None	None

*AE, adverse event; LCM, lacosamide; IgIV, intravenous immune globulin; KD, ketogenic diet; KET, ketamine; LEV, levetiracetam; MDZ, midazolam; PB, phenobarbital; PER, perampanel; SRSE, super-refractory status epilepticus; TPM, topiramate; VNS, vagus nerve stimulation; VPA, valproate*.

## Case #1

This was a female patient who was aged 16 years at the time of our observations. She had an unremarkable medical history until she experienced her first focal tonic seizure at the age of 8. Brain magnetic resonance (MR) at onset was normal, and electroencephalogram (EEG) showed bilateral temporal and occipital epileptiform abnormalities. She started valproate and clobazam. She was seizure-free for 4 years. At the age of 12, asymmetrical tonic-clonic seizures recurred despite conventional ASMs (lacosamide, primidone, and clonazepam). Seizures occurred weekly from ages 12 to 16, with some intermittent reduction in seizure frequency when a new ASM was added.

**Figure 1:**
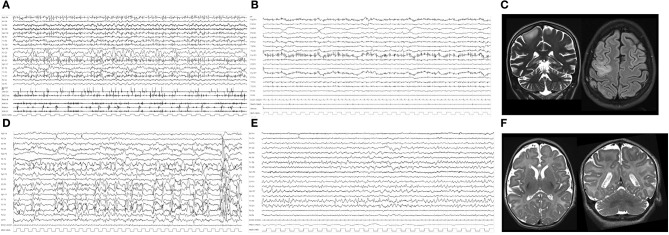
**(A)** Polygraphic video-EEG recording of Case #1, showing repetitive and continuous myoclonic jerks with an EEG counterpart characterized by diffuse repetitive spikes and multiple artifacts related to continuous myoclonic jerks of the face muscles. **(B)** Polygraphic recording after VNS implantation and after remission of myoclonic SE, showing low-voltage activity and rare myoclonic jerks on the EMG channels. **(C)** Coronal T2 and axial FLAIR MRI of Case #1 during RSE, showing mild cerebral atrophy, cerebellar atrophy, and hyperintensity over the right frontal region. **(D)** EEG during SE showing continuous epileptiform abnormalities over the left hemisphere and multiple repetitive spikes of Case #2. **(E)** EEG from 15 days after VNS implantation: epileptiform abnormalities were significantly reduced; some spikes over left temporal regions are evident. **(F)** Axial and coronal T2 brain MR showing cerebral atrophy and a simplification of cortical gyri, more evident over bilateral frontal and central regions of Case #2.

She came to our Department of Neuroscience with frequent myoclonic jerks involving the left side of the body, turning to super refractory myoclonic status. She was admitted to the Intensive Care Unit (ICU), and barbiturate coma was induced. First- and second-line treatments for SE and anesthetics were all ineffective. EEG recordings were consistently characterized by continuous epileptiform discharges over the right fronto-central area with bilateral diffusion (Figure 1A). Brain MR showed a hyperintensity on fluid-attenuated inversion recovery (FLAIR) sequences involving the right frontal region (Figure 1C). Metabolic workup was negative. Epilepsy genetic panel revealed a pathogenic heterozygous genetic variant in *ADCK3*.

Twenty-five days after admission, VNS (Demipulse 103 Cyberonics) was implanted without changes in ASM regimen (see Table 1). SRSE remitted after 7 days and did not recur (Figure 1B). Midazolam and phenobarbital doses were progressively reduced until they were stopped at 10 and 15 days, respectively, after VNS implantation. After VNS implantation, this patient presented daily myoclonic seizures that did not interfere with vital parameters. The patient died due to the underlying disease (dilated cardiomyopathy with a progressive reduction of ejection fraction until cardiac death) 5 months after VNS implantation at 17 years old.

## Case #2

This was the first daughter of healthy unrelated parents. Pregnancy and delivery were uneventful. Focal to bilateral tonic-clonic seizures associated with apnea and cyanosis lasting 30 s started at the age of 3.5 months. Interictal EEG showed slow and multifocal epileptiform abnormalities. Ictal EEG revealed diffuse low-voltage fast activity. Seizures were resistant to multiple ASMs (pyridoxin, phenobarbital, carbamazepine, phenytoin, clonazepam, and topiramate). From onset, she continued to present focal to bilateral tonic-clonic seizures every 4–8 days. She had profound intellectual disability, acquired microcephaly, and hypotonia with tetraplegia. Brain MR showed bilateral frontal simplification of cortical gyri at the age of 2 months, together with progressive diffuse cerebral atrophy at the age of 8 months (Figure 1F). Extended genetic epilepsy panels detected a *de novo* pathogenetic variant in *BRAT1* gene.

When aged 6 months, a refractory convulsive SE occurred (see Table 1). During this status, ASM and anesthetics were administered without success. On the 58th day after SRSE onset, VNS (Demipulse 103 Cyberonics) was implanted. SE remitted after 10 days and did not recur (Figures 1D,E). After VNS implantation, this patient presented weekly focal seizures that did not interfere with vital parameters. The patient died due to pediatric acute respiratory distress syndrome (P-ARDS) at the age of 3.

## Discussion

The usefulness of VNS implantation during RSE has been documented by anecdotal cases and in systematic reviews (3–6). Evidence supporting its efficacy is low (level IV), however, and the risk from reporting bias is high (4). We successfully implanted VNS into two patients with documented genetic epilepsy and SRSE. Previous evidence shows the median duration of RSE/SRSE pre- and post-VNS implantation to be 18 (range: 3–1,680) and 8 days (range 3–84), respectively (4). Consistent with previous reports, our patients were implanted 25 (#1) and 58 (#2) days after SE onset and, without ASM changes, a stable remission of SE was observed after the seventh (#1) and tenth (#2) day from VNS implantation. Significant positive effect has been reported in patients with both focal and generalized SE: cessation of SE was reported after a range of 3–14 days in generalized SE and between 15 and 60 days in focal SE (3). Death might occur during RSE, so aggressive treatment is often suggested. In one systematic review, four deaths (11%) during vagal stimulation for RSE/SRSE were reported, and all cases were considered unrelated to VNS implantation (4). In another systematic review of VNS, two out of the 28 patients reported died specifically during SE (3). Finally, in a patient recently described with Lafora disease, death occurred 9 months after implantation due to a tracheostomy complication (5). Our patients died due to the underlying disease (dilated cardiomyopathy with a progressive reduction of ejection fraction until cardiac death) in Case #1 and due to pediatric respiratory complications (P-ARDS) in Case #2.

The anti-seizure effect with VNS is, at least in part, time-dependent. However, the precise mechanism of the VNS anti-seizure effect, either acute or chronic, has not been fully elucidated. The Noda epileptic rat (NER) is a genetic epilepsy model that exhibits spontaneous generalized tonic-clonic seizure (GTC), approximately once every 30 h, and frequent dialeptic seizure (DS). Acute VNS in the NER significantly reduced the frequency of GTC and the duration of DS; chronic VNS decreased the frequency and duration of DS, but not GTC frequency, in a time-dependent manner. The brainstem and midline thalamus of NER were activated after acute and chronic VNS (7). VNS was also effective in the epileptic baboon, a naturally occurring animal model for genetic generalized epilepsy (GGE) (8).

VNS effectiveness in genetic epilepsies has previously been documented in various case series. In *CHD2* genetic epilepsy, VNS was associated with a good outcome, though secondary hemophagocytic lymph histiocytosis (SHLH) after a VNS wound infection was also reported (9). VNS was reported to be effective in patients carrying *CDKL5* genetic variants. One year after VNS insertion, 9/12 patients reported improved health-related quality of life (HRQoL) and 9/11 patients had improvements in mood, school achievement, and concentration (10). Similarly, HRQoL was improved following VNS by other authors in a *CDKL5* patient, and those authors concluded that adjunctive VNS therapy may widen the scope of treatment choices available to these patients, though they also acknowledged that the efficacy of VNS therapy for patients with intractable epilepsy associated with a genetic anomaly remains not fully established (11). Similar results have since been reported in genetic epilepsy with febrile seizures plus (GEFS+) and Dravet syndrome patients. In both of these, VNS seemed to exert a beneficial role in both seizure reduction and cognitive functions (12, 13).

Acute implantation of VNS during SRSE seemed to be effective in stopping SE in previous reports. Four cases have recently been reported with patients presenting different conditions: hemimegaloencephaly, non-ketotic hyperglycinemia, migrating focal seizures of infancy, and microdeletion of 1q43q44 causing microcephalia, corpus callosum agenesia, and epilepsy. All four conditions were genetically determined, and the authors reported cessation of SE in three out of the four cases (14).

Genetic testing has become more widespread, and more genetic mutations in patients with epilepsy are being identified; hence, previous reports of patients with RSE treated with VNS may have represented carriers of an unidentified, unconfirmed genetic mutation. We recognize that our patients are not unique in having genetic epilepsy, SRSE, and VNS; however, our experience does add to the data on confirmed genetic epilepsy.

The side-effect profile associated with VNS includes pharyngeal dysesthesias, change in voice, dysphagia, brady-arrhythmias, and asystole (15). We used a fast ramp-up of stimulations without evident side effects, even when patients were sedated, and this may have biased evaluation of adverse events.

For both of our patients, SE was super refractory—all medications, including anesthetics, were ineffective, as was the ketogenic diet tried in one case. We promptly considered alternative treatments; however, traditional surgery was not possible considering the genetic etiology of the epilepsies. VNS implantation for both patients was proposed by neurologists during epilepsy surgery meetings. The decision was driven by the general condition of the patients, which worsened more rapidly in Case #1 (implantation after 25 days from SE onset) than in Case #2 (implantation after 58 days from SE onset). Corpus callosotomy was another possible palliative surgical approach, which seems to be effective in refractory generalized epilepsy (16), though VNS was considered as the prior option within the treatment paradigm. (17, 18).

Multiple hypotheses exist regarding VNS in the treatment of epileptic seizures, including neurotransmitter modification, increase in the activity of the nucleus tractus solitarius, and a direct effect on neuronal desynchronization (19–21). The only hypothesis so far for explaining the efficacy during SE relies on single-photon emission computed tomography (SPECT) studies that show a normalization of cortical GABA_A_ receptor density (21). To date, despite encouraging evidence, the use of VNS has not been widely adopted for RSE.

We cannot know, either in the presented cases or in those previously reported, whether SE might have ceased independently of VNS implantation. However, we can speculate that both of our patients, who were experiencing SRSE without any reduction in the burden of epilepsy after several medications, were successfully treated with acute VNS. It is also worthwhile to underline that the majority of studies available are heterogeneous and retrospective, as well as that the use of VNS in RSE remains experimental.

Our results further suggest that VNS implantation should be considered as a safe and effective adjunctive treatment of RSE/SRSE when standard ASMs have failed and surgery is not possible. We acknowledge limitations in the present report, as well as in previously published case series reporting the effectiveness of VNS in RSE and SRSE, particularly the open-label approach and the limited number of cases published. Prospective studies should be designed to better understand the effectiveness of VNS in patients with SE.

## Data Availability Statement

The raw data supporting the conclusions of this article will be made available by the authors, without undue reservation, to any qualified researcher.

## Ethics Statement

Written informed consent was obtained from the patient for the publication of this case report.

## Author Contributions

NS and AF drafted the manuscript. NP and MT followed up the patients and reviewed the final draft of the manuscript. CC and GC drafted the figure, performed the EEGs and reviewed the final draft of the manuscript. AD and CM operated the patients, followed-up the VNS parameters and contributed to drafting the manuscript and approved the final draft of the manuscript. LP and FV coordinated the group and reviewed the final draft of the manuscript.

## Conflict of Interest

The authors declare that the research was conducted in the absence of any commercial or financial relationships that could be construed as a potential conflict of interest.
